# Effects of dihydropyridine calcium channel blockers on oxidized low-density lipoprotein induced proliferation and oxidative stress of vascular smooth muscle cells

**DOI:** 10.1186/1756-0500-5-168

**Published:** 2012-07-06

**Authors:** Jun Zou, Yan Li, Hong-Qi Fan, Ji-Guang Wang

**Affiliations:** 1 Centre for Epidemiological Studies and Clinical Trials The Shanghai Institute of Hypertension, Ruijin Hospital, Shanghai Jiaotong University School of Medicine, Ruijin 2nd Road 197, Shanghai, 200025, China

**Keywords:** Calcium channel blockers, Oxidative stress, Oxidized low-density lipoprotein, Proliferation, Vascular smooth muscle cells

## Abstract

**Background:**

Dihydropyridine calcium channel blockers (CCBs) are more effective in reducing carotid intima-media thickness (IMT) than other classes of antihypertensive drugs due to their vascular effects. However, the mechanism remains to be elucidated.

**Findings:**

Ox-LDL induced HUVSMCs proliferation in a time- and dose-dependent manner. When pretreated with three CCBs before 50 μg/ml ox-LDL stimulation, 30 μM lacidipine and 3 μM amlodipine exhibited 27% and 18% decrease of pro-proliferative effect induced by ox-LDL, whereas (*S*-)-amlodipine did not have any anti-proliferative effect. 30 μM lacidipine inhibited about two-thirds of the ox-LDL induced ROS production in HUVSMCs, whereas amlodipine and (*S*-)-amlodipine did not have influence on ROS production. The MAPKs pathway inhibitors inhibited the ox-LDL induced proliferation of HUVSMCs.

**Conclusion:**

Our study has demonstrated that lipophilic CCBs, such as lacidipine may inhibit ox-LDL induced proliferation and oxidative stress of VSMCs, and that the ROS-MAPKs pathway might be involved in the mechanism.

## Findings

In our previous meta-analysis, we found that calcium channel blockers (CCBs), mainly the dihydropyridine type, were more effective in reducing carotid intima-media thickness (IMT) than other classes of antihypertensive drugs such as diuretics, β-blockers, and angiotensin-converting enzyme (ACE) inhibitors [1]. This advantage of CCBs might contribute to their superiority in the prevention of stroke that had been repeatedly shown in previous studies [2]. However, the mechanism for the vascular advantage of CCBs remains to be elucidated.

IMT is a measure of vascular hypertrophy, which is in part the consequence of media thickening. It is known that the oxidized low-density lipoprotein (ox-LDL) cholesterol may induce oxidative stress of vascular smooth muscle cells (VSMCs) by activating the mitogen-activated protein kinases (MAPKs) pathway, and in turn promote proliferation of VSMCs [3,4] and hence vascular hypertrophy. The oxidative mechanism may increase formation of intracellular reactive oxygen species (ROS), which may behave as intracellular second messengers and modulate expression of genes that influence the development of vascular lesions [5]. We hypothesized that CCBs might exert their vascular protective effect via the oxidative stress pathway in VSMCs.

Several long-acting CCBs have been studied for their vascular effects, and are frequently used in clinical practice in many countries, such as amlodipine [6], lacidipine [7] and azelnidipine [8]. These long-acting CCBs are indifferent in the blood pressure lowering effect, but different in lipophilicity and hence the binding affinity with the calcium channels inside the lipophilic cell membrane. Lacidipine is highly lipophilic and has long-term blood pressure lowering effect, even though its terminal elimination half-life (t_1/2z_) is approximately 6–10.8 h [9]. The lipophilic property of lacidipine can also be important in vascular protection, because it apparently may interact with cells in the absence of L-type calcium channels, and give lacidipine vascular protection effects independent of its actions on blood pressure [9]. Amlodipine is also lipophilic [7] but less than lacidipine [9]. Amlodipine has two enantiomers. It is known that only the (*S*-)-isomer of amlodipine has blood pressure lowering effect, and has recently been available in the Chinese market as a separate drug from amlodipine. However, there is some evidence that the (*R*+)-isomer of amlodipine is not a simple bystander, but might exert vascular protective effect, and play a role in the peculiar protective effect of amlodipine [10,11], as repeatedly shown in large randomized controlled trials in hypertension [2] and coronary artery disease [7]. We therefore investigated the effect of lacidipine, amlodipine and (*S-*)-amlodipine on the ox-LDL induced proliferation and oxidative stress of VSMCs.

## Results

### Pro-proliferation of ox-LDL

**Figure 1:**
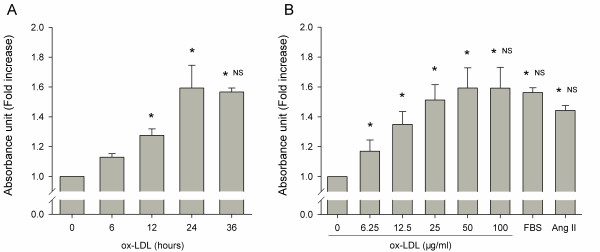
**Time-course and dose-dependent effects of ox-LDL on HUVSMCs proliferation.** During the experiment, medium with 0.4% FBS was employed. At the end of each experiment, a water-soluble tetrazolium salts (WST-1) assay was performed to quantify the number of proliferation cells. (**a**). Cells incubated with ox-LDL (50 μg/ml) in various periods. * P < 0.05 vs. corresponding 0 h control. NS indicates no significance vs. 24 h incubation of ox-LDL. (**b**). Cells exposed to various concentrations of ox-LDL, FBS (5%) or Ang II (10^-7^ M) as indicated. * *P* < 0.05 vs. corresponding no ox-LDL control. NS indicates no significance vs. ox-LDL (50 μg/ml). Data are mean ± SD of three to six independent experiments determined in duplicate, and expressed as fold increase of absorbance in 440 nm compared with unstimulated cells.

To determine the right time and dosage of ox-LDL, we examined effects induced by ox-LDL on various incubation time and dosages. After 6, 12, 24 and 36 h of incubation with ox-LDL (50 μg/ml), quiescent HUVSMCs were stimulated proliferation in a time-course manner (Figure 1a). Ox-LDL exerted pro-proliferative effects, at 12 h of incubation, and the maximal effect at 24 h of incubation. After 24 h of incubation with various concentrations of ox-LDL (6.25-100 μg/ml), FBS (5%) or Ang II (10^-7^ M), HUVSMCs were stimulated proliferation with ox-LDL also in a dose-dependent manner (Figure 1b). Ox-LDL began to manifest significant mitogenic effects at 6.25 μg/ml, and the maximal effect at 50 μg/ml. The maximal pro-proliferative effect of ox-LDL after 24 h of incubation and at 50 μg/ml of dosage was an approximately 60% (1.59 ± 0.13 fold) increase in the proliferation of HUVSMCs, as compared with that of unstimulated cells. This effect was similar to FBS (1.56 ± 0.03) and slightly greater than Ang II (1.44 ± 0.03, *P* = 0.06). Further increasing time and concentrations of ox-LDL co-incubation exerted cytotoxic or apoptotic effects on VSMCs (data not shown).

### Effects of CCBs on cell proliferation

**Figure 2:**
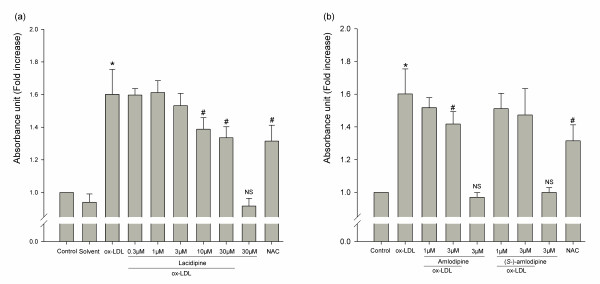
**Effects of three lacidipine (a), amlodipine and (S-)-amlodipine (b) and NAC on ox-LDL induced HUVSMCs proliferation.** Cells were pretreated for 1 h with or without CCBs or with NAC (5 mM), and then treated with or without ox-LDL (50 μg/ml). Data are mean ± SD of five to eight independent experiments determined in duplicate (three independent experiments for (*S-*)-amlodipine), and expressed as fold increase of absorbance in 440 nm compared with unstimulated cells. * *P* < 0.05 vs. corresponding no ox-LDL control. # *P* < 0.05 vs. ox-LDL. NS indicates no significance vs. corresponding solvent group (**a**) or vs. the control group (**b**).

We then studied effects of three CCBs (lacidipine, amlodipine and (*S*-)-amlodipine) on the ox-LDL induced HUVSMCs proliferation. Cells were pretreated for 1 h with the three CCBs or NAC (N-acetyl-L-cysteine, intracellular reactive oxygen species scavenger) before the 24 h of incubation with ox-LDL (50 μg/ml). 10 μM and 30 μM lacidipine exhibited 21% (*P* < 0.001) and 27% (*P* < 0.001) decreases of the pro-proliferative effect induced by ox-LDL, respectively, and the anti-proliferative effect was dose-dependent (*P* < 0.001, Figure 2a). 3 μM amlodipine exhibited 18% (*P* = 0.01) decrease, while (*S*-)-amlodipine did not exhibit any anti-proliferative effect in our experimental conditions at a concentration equal to amlodipine (Figure 2b). 5 mM NAC exhibited approximately a 28% (*P* < 0.001) decrease. These CCBs at the dosages shown in Figure 2 did not show significant effect on cell viability except for 30 μM lacidipine on which cell viability was significantly different from the control group (*P* = 0.02) but not from the solvent group (*P* = 0.39). However, if further increasing the dosage of three CCBs, cell viability significantly decreased (data not shown).

### Effects of CCBs on cell ROS production

**Figure 3:**
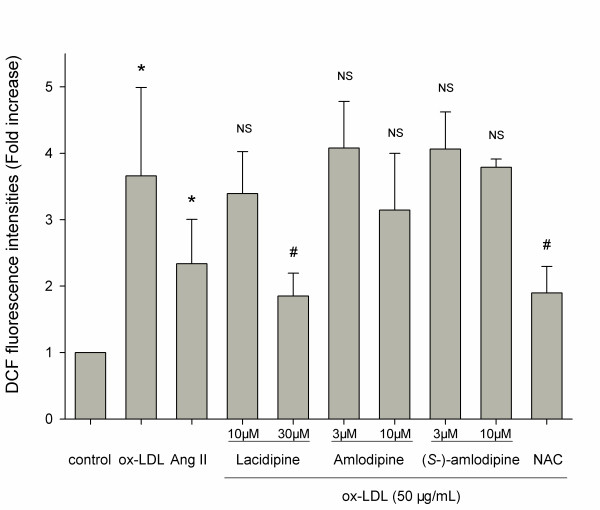
**Ox-LDL and Ang II induced ROS generation in HUVSMCs and effects of three CCBs (lacidipine, amlodipine and (*S-*)-amlodipine) and NAC.** Cells were pretreated for 30 min with or without CCBs or with NAC (5 mM), and then treated with Ang II (10^-7^ M) or ox-LDL (50 μg/ml) for 1.5 h. Then, the DCFH-DA (10 μM) probe was added and incubated away from light for another 30 min. Thereafter, cells were immediately washed twice with PBS, and added to 400 μL DMEM, and the fluorescent intensity was read five times and averaged. The excitation wavelength is 504 nm, and the emission wavelength is 529 nm. Data are mean ± SD of five to eight independent experiments determined in duplicate (three independent experiments for (*S-*)-amlodipine), and expressed as fold increase of DCF fluorescence intensities compared with unstimulated cells. * *P* < 0.05 vs. corresponding no ox-LDL control. # *P* < 0.05 vs. ox-LDL. NS indicates no significance vs. ox-LDL.

DCF (2′,7′ -dichlorofluorescein) production, detected by a fluorescence microplate reader and expressed as fold increase of fluorescence intensities compared with unstimulated cells, represented the intracellular ROS generation. Figure 3 shows that DCF production of HUVSMCs induced by the incubation with ox-LDL (50 μg/ml) for 2 h tended to be greater (*P* = 0.07) than that by the incubation with Ang II (10^-7^ M). When pretreated for 30 min with these three CCBs and NAC, only 30 μM lacidipine (*P* = 0.02) and 5 mM NAC (*P* = 0.03) exerted inhibition. Lacidipine inhibited about two-thirds (*P* = 0.02) of the ox-LDL induced DCF production in HUVSMCs, with a potency similar to NAC. 10 μM amlodipine inhibited about one-fifth (*P* = 0.46), and (*S*-)-amlodipine did not inhibit the ox-LDL induced intracellular increase of DCF production.

### Effects of MAPKs inhibitors on cell proliferation

**Figure 4:**
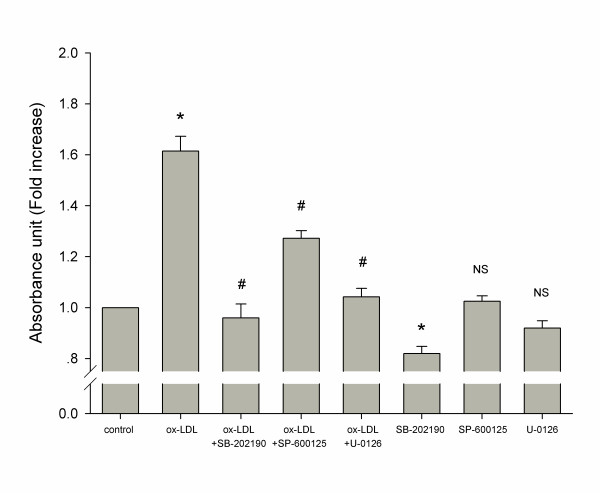
**Involvement of the MAPKs signaling pathway in the effect of ox-LDL on HUVSMCs.** Cells were pretreated for 1 h with or without inhibitors of the MAPKs signaling pathway: SB-202190 (10 μM, p38 MAPK inhibitor), SP-600125 (10 μM, JNK inhibitor) and U-0126 (10 μM, MEK inhibitor), and then treated with or without ox-LDL (50 μg/ml). Data are mean ± SD of four independent experiments, and expressed as fold increase of absorbance compared with unstimulated cells. * *P* < 0.05 vs. control. # *P* < 0.05 vs. ox-LDL. ** *P* < 0.05 vs. control. NS indicates no significance vs. control.

It is known that the MAPKs signaling pathway is involved in the proliferation of VSMCs. Three MAPKs pathway inhibitors, SB-202190 (10 μM, p38 MAPK inhibitor), SP-600125 (10 μM, JNK inhibitor) and U-0126 (10 μM, MEK inhibitor), were used as tool drugs to determine whether the MAPKs pathway was involved in the ox-LDL induced proliferation on HUVSMCs. Cells were pretreated with the tool drugs for 1 h, and then incubated with ox-LDL (50 μg/ml) for 24 h. All these three tool drugs inhibited (35%–66% decrease, *P* < 0.001) the ox-LDL induced proliferation of HUVSMCs (Figure 4). SB-202190 had the strongest effect of inhibition. In the absence of ox-LDL, only SB-202190, but not the other tool drugs, showed a significant effect of inhibition in comparison with control.

## Discussion

Our study has demonstrated an anti-proliferative and anti-oxidative effect of the lipophilic CCBs on HUVSMCs. The most lipophilic CCB lacidipine may inhibit proliferation and oxidative stress of VSMCs. Amlodipine also inhibits proliferation but does not influence oxidative stress, whereas its (*S*-)-isomer, (*S*-)-amlodipine, does not have anti-proliferative, nor anti-oxidative effect. Our finding may have clinical implications, because enhanced proliferation of VSMCs and change from the contractile to proliferative/secretory phenotype may contribute to the pathogenesis of atherosclerosis, restenosis after angioplasty, and graft atherosclerosis after coronary transplantation [12,13], and because intracellular ROS regulates phenotypic differentiation of VSMC [14].

Similar anti-proliferative and anti-oxidative effects of lacidipine had been observed on other cell lines. In human umbilical vein endothelial cells, lacidipine might decrease NF-κB-mediated and ox-LDL induced adhesion molecule expression [15]. This effect of lacidipine might be the consequence of its influence on the intracellular ROS formation induced by ox-LDL [16].

In keeping with several previous studies [17-19], our study showed that amlodipine might also inhibit VSMC proliferation. However, we only observed a weak and nonsignificant anti-oxidative effect of amlodipine, which was different from the results of previous studies [19,20]. Other mechanisms than the anti-oxidative effect might be involved in the anti-proliferative effect of amlodipine, such as, calcium signaling through ERK1/2 cascade, nitric oxide (NO) production, modulation of matrix metalloproteinases activity, etc. [19].

At least one previous study suggested that the vascular effect of amlodipine was attributable to the action of its (*R*+)-isomer, which had no effect on blood pressure [10]. In endothelial cells, which had no calcium channels, (*R*+)-amlodipine, but not (*S*-)-amlodipine might activate NO synthase and promoted the production of NO [10,11]. In our study in VSMCs, (*S-*)-amlodipine did not inhibit ox-LDL induced proliferation and oxidative stress of HUVSMCs. The distinct effect of amlodipine and (*S*-)-amlodipine is not understood. It is possible that (*R*+)-amlodipine and (*S*-)-amlodipine have different lipophilicities, and hence have different vascular effects. Taking into account the strong effect of the highly lipophilic CCB lacidipine, this explanation is very likely.

We also studied the role of ROS in the proliferation of VSMCs by the use of NAC, a ROS scavenger, and the role of the MAPKs signaling pathway by the use of the distinct inhibitors of this pathway. Our finding of these experiments suggested that the ROS-MAPKs pathway might be one of mechanisms for the effect of CCBs on proliferation of VSMCs. This mechanism may also explain why lacidipine has strong anti-proliferative effect. The dihydropyridine ring of the higher lipophilic CCBs, such as lacidipine, facilitates proton-donating and resonance-stabilization mechanisms that quench the free radical reaction, thereby blocking the peroxidation process and ROS production [21].

## Conclusion

In conclusion, our study has demonstrated that lipophilic CCBs, such as lacidipine may inhibit ox-LDL induced proliferation and oxidative stress of VSMCs. Although our finding is in line with the results of previous animal studies [22,23] and human research [6,24] on the vascular effects of CCBs, further studies in animals and humans are still required to compare these long-acting CCBs at similar blood pressure lowering dosages.

## Methods

### Chemicals and reagents

In our experiments, three CCBs were employed. Lacidipine (courtesy of GlaxoSmithKline China, Shanghai, China) was dissolved in the mixture of Tween-80 and ethanol (2:8). Amlodipine (a 1:1 mixture of two enantiomers; courtesy of Pfizer China, Shanghai, China) and (*S-*)-amlodipine (courtesy of Shihuida, Changchun, China) were all dissolved in dimethyl sulfoxide (DMSO). Ox-LDL (about 1.6 mg/ml) was purchased from UnionBiol (Beijing, China), and the extent of the LDL oxidation was determined by measuring the levels of thiobarbituric acidreactive substances (TBARS). SB-202190 (an inhibitor of p38 MAPK), SP-600125 (an inhibitor of JNK) and U-0126 (an inhibitor of MEK) were purchased from CalBiochem (San Diego, California, USA). The following chemicals were purchased from Sigma-Aldrich (Shanghai, China): Angiotensin II, NAC, 2′, 7′-dichlorofluorescin diacetate (DCFH-DA), DMSO and Tween 80. We also purchased cell proliferation reagents WST-1 (Roche, Basel, Switzerland), fetal bovine serum (FBS; Invitrogen, California, USA) and Typan Blue (Beyotime, Jiangsu, China).

### Cell culture

Human umbilical vein smooth muscle cells (HUVSMCs) were obtained from the Health Protection Agency Culture Collections (Porton Down, Salisbury, UK). Cells were grown in Dulbecco’s Modified Eagle Medium (DMEM) of high glucose supplemented with 10% FBS without antibiotics. The cells were maintained 37°C in a 5% CO_2_ incubator. The medium was changed every 2 days. Cells were digested with 0.25% trypsin and 0.02% EDTA when cells reached 70-80% confluency, and were serum-deprived in DMEM with 0.1% FBS for 24 h before each experiment when cells reached 60-70% confluency. After serum deprivation, the cells were treated with reagents, pharmacological inhibitors and treatment drugs as noted in the figure legends. During the period of experiment, medium with 0.4% FBS was employed for the maintenance of cell viability. Trypan blue exclusion test was applied to detect cell viability. On our experimental conditions, cell viability after each experiment was always greater than 95%.

### Cell proliferation assay

In order to determine the effect of a compound on cell growth, serum-starved cells at a density of 1 × 10^4^ cells/well were incubated on 24-well plates for 24 h followed by another 2.5 h of incubation with water-soluble tetrazolium salts (WST-1). The tetrazolium salts were cleaved to soluble formazan by intracellular mitochondrial dehydrogenases in the sample. This augmentation in enzyme activity led to an increase in the amount of formed formazan dye, which was directly correlated with the number of proliferative cells in the culture. Quantification of the formazan dye produced by metabolically active cells was measured at 440 nm wavelengths by scanning the multiwell spectrophotometer. Because various concentrations of 2 different solvents were used for these experiments, we set a solvent-only control group for each of the 2 different solvents at higher concentrations.

### Detection of intracellular ROS

DCFH-DA is a dye that allows monitoring of ROS by the fluorescence microplate spectrophotometer. This method is based on the oxidation of DCFH-DA by ROS, resulting in the formation of the fluorescent compound DCF. Serum-starved cells in 24-well plates were preincubated with the three CCBs and NAC for 30 min as described above, and ox-LDL (50 μg/ml) was then added to the medium for 1.5 h. DCFH-DA (10 μM) probe was then added and incubated for another 30 min. Thereafter, cells were immediately washed twice with PBS and added to 400 μl DMEM. The fluorescent intensity was read five times and averaged. The excitation wavelength was 504 nm, and the emission wavelength was 529 nm.

### Statistical analysis

Values were mean ± SD. Means were compared by one-way ANOVA with the *post hoc* test for multiple comparisons (Dunnett’s test for comparison of a single control or Bonferroni correction for pairwise comparisons). *P* < 0.05 was considered statistically significant.

## Competing interests

Dr Wang reports receiving consulting and lecture fees from Astra-Zeneca, Bayer, GSK, Omron, Pfizer, Sankyo, Sanofi, and Servier. The other authors report no conflicts of interest. The authors alone are responsible for the content and writing of the paper.

## Authors’ contributions

JGW designed the study, guided the experiments, contributed to the analysis and interpreted the results. JZ conducted the experiments, participated in the analysis and interpretation and drafted the manuscript. YL guided the experiments and was involved in drafting the manuscript. HQF participated in the study design development. All authors have read and approved the final manuscript.
